# Importance of the *Aspergillus fumigatus* Mismatch Repair Protein Msh6 in Antifungal Resistance Development

**DOI:** 10.3390/jof10030210

**Published:** 2024-03-12

**Authors:** Jose Lucio, Irene Gonzalez-Jimenez, Alejandra Roldan, Jorge Amich, Laura Alcazar-Fuoli, Emilia Mellado

**Affiliations:** 1 Mycology Reference Laboratory, National Centre for Microbiology, Instituto de Salud Carlos III (ISCIII), 28220 Majadahonda, Spain; joslucio2@gmail.com (J.L.); alex7799roldan@gmail.com (A.R.); jamich@isciii.es (J.A.); lalcazar@isciii.es (L.A.-F.); 2 CIBER de Enfermedades Infecciosas (CIBERINFEC-CB21/13/00105), Instituto de Salud Carlos III (ISCIII), 28029 Madrid, Spain

**Keywords:** *Aspergillus fumigatus*, antifungal resistance, *msh*6, MMR pathway

## Abstract

One of the systems responsible for the recognition and repair of mistakes occurring during cell replication is the DNA mismatch repair (MMR) system. Two major protein complexes constitute the MMR pathway: MutS and MutL. Here, we investigated the possible relation of four *A. fumigatus* MMR genes (*msh*2, *msh*6, *pms*1, and *mlh*1) with the development of azole resistance related to the phenomenon of multi-drug resistance. We examined the MMR gene variations in 163 *Aspergillus fumigatus* genomes. Our analysis showed that genes *msh*2, *pms*1, and *mlh*1 have low genetic variability and do not seem to correlate with drug resistance. In contrast, there is a nonsynonymous mutation (G240A) in the *msh*6 gene that is harbored by 42% of the strains, most of them also harboring the TR_34_/L98H azole resistance mechanism in *cyp*51A. The *msh*6 gene was deleted in the *aku*B^KU80^ *A. fumigatus* strain, and the ∆*msh*6 isolates were analyzed for fitness, azole susceptibility, and virulence capacity, showing no differences compared with the *aku*B^KU80^ parental strain. Wild-type *msh*6 and Δ*msh*6 strains were grown on high concentrations of azole and other non-azole fungicides used in crop protection. A 10- and 2-fold higher mutation frequency in genes that confer resistance to boscalid and benomyl, respectively, were observed in Δ*msh*6 strains compared to the wild-type. This study suggests a link between Msh6 and fungicide resistance acquisition.

## 1. Introduction

Triazoles are the main drugs used for treatment and prophylaxis against *Aspergillus* spp. infections [1,2]. However, the increased worldwide detection of azole-resistant *Aspergillus fumigatus* isolates is threatening azole drugs’ effectiveness in aspergillosis management [3]. Triazole drugs belong to the demethylation inhibitors drug family (DMIs), which targets fungi at the 14-α sterol demethylase (Cyp51), an essential enzyme from the ergosterol biosynthesis route [4]. In the clinical setting, the four triazole drugs used as first-line antifungal treatment are mainly voriconazole and isavuconazole, and also itraconazole and posaconazole. On the other hand, in the agricultural environment, DMIs are used to protect crops against different fungal plant pathogens, but due to their chemical structure similar to clinical azoles, cross-resistance between both drug families is common [5,6,7]. In addition, other classes of fungicides, such as benzimidazoles (MBCs), strobilurins (QoIs), and succinate dehydrogenase inhibitors (SDHIs), are also intensively used to protect crops, together with DMIs, creating an environmental pressure to the evolution of resistance against all major classes of fungicides [8].

The development of azole resistance in *A. fumigatus* occurs due to a selective pressure of antifungals that can be applied in two different scenarios: the clinical path, usually as a result of long periods of exposure to azole clinical therapy, which associates with single point mutations in *cyp*51A, and an environmental acquisition due to the use of DMIs in the agricultural setting, related to a combination of Cyp51A modifications (several *cyp*51A point mutations together with tandem repeat insertions (TR), in the promoter of *cyp*51A) [9]. The majority of the azole-resistant isolates described to date have been associated with the environmental route, whereas the resistant isolates developed in clinics have arisen after long azole treatments [10]. In plant pathogens, DMI resistance shows a wide variety of mechanisms, although the combination of Cyp51 point mutations and promoter insertions is predominant among diverse fungal species [9].

Unlike DMIs, the impact of non-azole pesticides in the development of *A. fumigatus* azole resistance has been poorly studied [8,11,12]. Some of these non-azole fungicides mentioned previously, including QoIs, SDHIs, and MBCs, are widely used [13], and some studies have reported resistant strains harboring point mutations in their target genes. Resistance mechanisms to QoIs have been associated with point mutations in the cytochrome *bc*1 gene (*cyt*B) [14,15]; SDHIs resistance mechanisms have been related to point mutations in the *sdh*B subunit [15,16]; and lastly, point mutations in the β-tubulin gene (*ben*A) have been linked to resistance to MBCs [17]. We have recently used a collection of *A. fumigatus* strains to reveal a correlation between resistance to these fungicides and the azole resistance mechanisms involving TRs, showing a multi-resistance pattern phenomenon in *A. fumigatus* that could be related to their specific genetic background [8].

In previous whole genome sequencing (WGS) phylogenetic analysis performed in our lab, a genome collection of azole-susceptible and resistant *A. fumigatus* strains from diverse geographical origins was bundled into four different clusters. The most remarkable finding was that all genomes harboring the azole resistance mechanisms with TRs in *cyp*51A promoter were grouped in the same cluster [8,18], suggesting genetic closeness possibly due to additional genetic mechanisms operating in these genomes that could include fitter genotypes. Some studies have suggested genetic instability as a possible mechanism of evolving azole resistance in *A. fumigatus* [19,20], as well as in other fungal pathogens [21,22,23]. In this sense, the avoidance of mistakes during cell replication allows for genetic stability, which is key for the enduring survival of organisms [24]. To ensure genome stability and to sense and repair damaged DNA, cells use a global signaling network named the DNA damage response (DDR), which includes cell cycle checkpoints, chromatin remodeling, DNA repair (MMR), and DNA-damage tolerance pathways (DDT) [19,25]. Activation of DNA damage checkpoint is the core event of DDR, and in *S. cerevisiae*, this activation involves two highly conserved protein kinases, ScMec1 (ATM) and ScTel1 (ATR), that initiate evolutionarily conserved signal transduction cascades, and repair of DNA damage [25]. The role of these kinases in *A. fumigatus* has been previously investigated, suggesting that genetic instability caused by Δ*atm*A and Δ*atr*A mutations can confer an adaptive advantage, mainly in the intensity of voriconazole resistance acquisition [19].

The DNA mismatch repair (MMR) system conducts the recognition and repair of any mistakes occurring during DNA replication [26]. This pathway is constituted by two protein complexes: MutS, which recognizes and binds to the mismatch, and MutL, which interacts with the mistake and removes the strand [27]. Multiple homologs of these proteins have been identified among the eukaryotic microorganisms, best characterized in *Saccharomyces cerevisiae* [28]. Although the MMR has been poorly investigated in *A. fumigatus* compared to *S. cerevisiae*, the genes comprising it are well conserved through all phylum *Ascomycota*, including *msh*1, *msh*2, *msh*3, *msh*4, *msh*6, and *mlh*1 [29]. Defects in MMR, such as gene loss or alterations of the pathways, can cause alterations in the recognition and repair of mutations, creating, in this scenario, an increased mutation rate associated with mutator phenotypes [28,29,30].

In this study, we investigated four *A. fumigatus* MMR genes, *msh*2, *msh*6, *pms*1, and *mlh*1, to evaluate their possible implication in genetic instability and thus their potential contribution to the development of antifungal resistance by searching for mutations in these genes among a collection of environmental and clinical *A. fumigatus* isolates. In view of the results obtained, we deleted *msh*6 to evaluate the importance of genetic instability in *A. fumigatus* as a possible mechanism of evolving antifungal resistance. A comparison of mutation frequencies in *A. fumigatus* wild-type and Δ*msh*6 strains showed that the loss of Msh6 results in increased mutation frequencies when exposed to some antifungals, allowing for a rapid generation of resistance.

## 2. Materials and Methods

### 2.1. Aspergillus Fumigatus Strains and Culture

A total of 144 clinical *A. fumigatus* strains obtained over a period of 20 years (2001 to 2021) belonging to the Spanish National Center of Microbiology were used in this study. This collection comprises a heterogeneous group of isolates, including azole-susceptible and azole-resistant strains. Resistant strains include a selection of the most common azole resistance mechanisms described to date (G54, M220, G448S, TR_34_/L98H, TR_34_/L98H/S297T/F495I, TR_46_/Y121F/T289A, TR_53_). Species-level identification was performed by PCR amplification and sequencing of the ITS1-5.8S-ITS2 regions and a portion of the β-tubulin gene [31]. The reference strain *aku*B^KU80^ was used to perform all the transformation experiments.

The *A. fumigatus* strains were subcultured on potato dextrose agar (PDA) (Oxoid, Madrid, Spain) and incubated at 37 °C until the colonies were visually sporulated (48–72 h). For each experiment, conidia were freshly harvested from a PDA medium with 0.1% Tween 20 (Sigma-Aldrich, Madrid, Spain). The concentration of conidia was measured using hemocytometer counting. For long-lasting conservation, *A. fumigatus* spore suspensions were mixed with an equal volume of autoclaved 50% glycerol, and 0.5 mL aliquots of the mix were stored in cryogenic vials at −80 °C.

### 2.2. Whole Genome Sequencing Analysis

A search for variants or mutations in MMR genes was performed within a collection of 163 *A. fumigatus* genomes that had been previously sequenced in our laboratory using Nextera^®^ XT Library Prep Kit (Illumina Inc., San Diego, CA, USA) as described before or obtained from public databases [8].

The Illumina reads were trimmed using Trimmomatic (version 0.32) [32]. The sequencing adapters and sequences with low-quality scores on 30 ends (Phred score [Q], <20) were trimmed. Raw Illumina WGS reads were quality-checked by performing quality control with FastQC (version 0.11.3; Babraham Institute, Cambridge, UK). Data sets were analyzed against the *A. fumigatus* A1163 reference genome (GenBank accession number ABDB00000000.1) using WGS-Outbreaker v1.0 (Instituto de Salud Carlos III, Madrid, Spain) (https://github.com/BU-ISCIII/WGS-Outbreaker, accessed on 9 October 2018) with default parameters. The pipeline comprised all steps needed for single nucleotide variant (SNV) analysis using whole-genome sequencing data. Mapping against the genome reference was performed with bwa mem (version 0.7.12-r1039) [33], duplicated reads were removed using Picard (version 1.140) (http://broadinstitute.github.io/picard, accessed on 9 October 2018), and the bedtools coverage v2.26 program [34], was used to perform further quality controls. Here, in order to identify genetic variations among strains, SNV detection (variant calling) and SNV matrix generation were performed using GATK version 3.8.0 [35], with best-practices parameters. The ENSEMBL variant effect predictor script (version 88) was used for variant annotation. Part of the whole genome sequencing project has been deposited in NCBI SRA (project accession number SRP151231). All the data used in this analysis, including information about clinical antifungal susceptibility and azole resistance mechanisms of the strains, can be found in Supplementary Table S1.

### 2.3. Phylogenetic Analysis and Single-Nucleotide Variant Comparison

The final step of the WGS-Outbreaker pipeline comprised Maximum-likelihood tree construction using RaxML software (version 8.2.9) [36], with a GTRCAT model and 100 bootstrap replicates. Phylogenetic trees were visualized and annotated using the ggtree R package [37], and SNV comparisons were performed using a custom R script, mapping all genomes to the A1163 reference genome.

### 2.4. Generation of A. fumigatus Δmsh6 Strains

#### 2.4.1. Vector Construction

**Figure 1:**
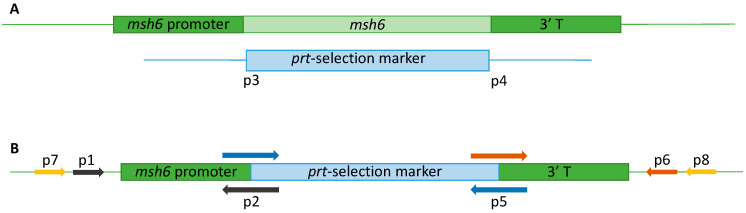
Construction of the fusion cassette for *A. fumigatus msh6* deletion in the *aku*B^KU80^ strain. The deleted *msh*6 gene is indicated in light green, and the pyrithiamine selection marker in light blue. (**A**) Map of the parental *msh*6-WT strain and the pyrithiamine (*prt*) selection marker. (**B**) Design of the fusion cassette for *A. fumigatus* Δ*msh*6 strain.

To assess the role of Msh6, the corresponding *msh*6 gene was deleted from the *A. fumigatus* strain *aku*B^KU80^ and replaced by the resistance marker pyrithiamine (*prt*) (Figure 1). The corresponding fusion cassette was constructed by overlapping PCR [38]. The PCR conditions for amplification of upstream, downstream, and the *prt* fragment excerpts were 2 min at 98 °C and then 30 cycles of 30 s at 98 °C, 15 s at 55 °C and 2 min at 98 °C, with a final elongation step of 7 min at 68 °C. The PCR reaction mix was performed using the PrimeSTAR^®^ HS DNA Polymerase (Takara Bio USA, Inc., San Jose, CA, USA) following the manufacturer’s instructions using primers p1 and p2 for the upstream excerpt, p4 and p6 for the downstream and p3 and p5 fo the *prt* insert (Table S2). For the amplification of the final fusion cassette, PCR conditions were 95 °C for 1 min, then 35 cycles of 95 °C for 30 s, followed by 6 min at 68 °C, ending with a final step of 6 min at 68 °C. Primers used for amplifying the whole cassette were p1 and p6 (Table S2). PCR was performed using the Advantage^®^ Polymerase Mix (Takara Bio USA, Inc.) following the manufacturer’s recommendations, mixing equal amounts of the three excerpts to conform the cassette. The primer locations and the fusion vector design are shown in Figure 1.

#### 2.4.2. Transformation

*Aspergillus fumigatus* transformation was achieved using protoplasts, as described before [39]. The resulting transformants were selected on minimal medium plates containing 0.5 g/mL pyrithiamine (Sigma Aldrich, Madrid, Spain). Mutants were confirmed by PCR and sequencing using primers p7 and p8 (Figure 1, Table S3), and the positive ones were named with a letter T (transformant) followed by a number.

### 2.5. Phenotypic Characterization of Δmsh6 Mutant

A total of 10^4^ conidia from two independent ∆*msh*6 strains (T3 and T6) and its parental WT strain (*aku*B^KU80^) were grown in solid MM containing 0.04 μM, 0.06 μM, and 1.6 μM of menadione; 0.005% and 0.02% of H_2_O_2_; 10 mg/L, 50 mg/L, and 100 mg/L of Congo Red (Sigma, Madrid, Spain); and 10 mg/L and 100 mg/L of Calcofluor white (Sigma, Madrid, Spain). Plates were incubated for 72 h at 37 °C, and at least two biological replicates were performed for each test.

### 2.6. Galleria Mellonella Survival Assay

To determine whether there were differences in virulence, the parental strain and the generated ∆*msh*6 (T3 and T6) mutant strains were tested in the alternative model of infection *G. mellonella*. Wax-moth larvae used (TruLarv™ BioSystems Technology, Exeter, UK) were infected with *A. fumigatus* mutants and Δ*aku*B^KU80^ strain as a control. Wax-moth larvae killing assays were performed as previously described [40]. Twenty larvae (0.2–0.3 g) were inoculated with 10^3^ and 10^4^ conidia per larvae for each strain and incubated at 37 °C for 8 days, during which mortality was recorded daily. Each experiment was performed at least 3 times, and results were reported as mean values. Statistical analyses were performed with the GraphPad Prism software package (version 11.0) (SPSS Inc., Chicago, IL, USA). Kaplan-Meier survival curves were analyzed by using a log-rank (Mantel-Cox) test for significance. A *p*-value < 0.01 was considered significant.

### 2.7. Antifungal Susceptibility Testing

Antifungal susceptibility tests were performed following the E-test method [41] for itraconazole (ITC), voriconazole (VCZ), and Posaconazole (PSZ) (bioMérieux, Madrid, Spain). An inoculum adjusted to 10^5^ conidia per ml was used, and 200 μL were plated in RPMI 1640 (Sigma-Aldrich Quimica SA, Madrid, Spain) agar base plates supplemented with 2% glucose. Minimal inhibitory concentrations (MICs) were read after 48 h incubation at 35 °C. Confirmation of antifungal susceptibility testing was performed using the broth microdilution method described by the European Committee on Antimicrobial Susceptibility Testing (EUCAST) [42]. The antifungal agents tested were ITC (Janssen Pharmaceutical, Madrid, Spain), VCZ (Pfizer, Madrid, Spain), PSZ (Schering-Plough, Madrid, Spain), and Amphotericin B (AmB) (Sigma, Madrid, Spain). *Aspergillus flavus* ATCC204304 and *A. fumigatus* ATCC204305 were used as quality control strains. In vitro susceptibility and resistance were defined according to the epidemiological cutoff values published for *A. fumigatus*. Isolates with ITC and VCZ MICs ≤ 1 μg/mL and PSZ ≤ 0.25 μg/mL were considered susceptible [43]. Both susceptibility tests were repeated at least twice for each strain.

### 2.8. Mutagenesis Experiments in ∆msh6 Strains

Putative gains of antifungal resistance were assayed by plating a total of 10^9^ conidia of two Δ*msh*6 null mutant strains (T3 and T6) and their parental WT strain (*aku*B^KU80^) on solid minimal media (MM) supplemented with one of the following: 2 mg/L of PSZ, 4 mg/L of VCZ, 32 mg/L of prochloraz (PRZ), benomyl (BNY), boscalid (BCL), or azoxystrobin (AZB). All plates were incubated at 37 °C for up to 14 days until resistant colonies emerged. Colonies were picked and streaked in a new MM plate supplemented with the same antifungal at the matching concentration. Strains that grew again in these plates were picked and isolated for further genetic analysis.

### 2.9. Genetic Analysis of the Mutants

Genomic DNA was extracted from all *A. fumigatus* mutants, followed by PCR amplification and sequencing of *sdh*B, *ben*A, and *cyp*51A genes, as previously described [8,44].

## 3. Results

### 3.1. Analysis of Mutations in MMR Genes in a Collection of Isolates

**Figure 2:**
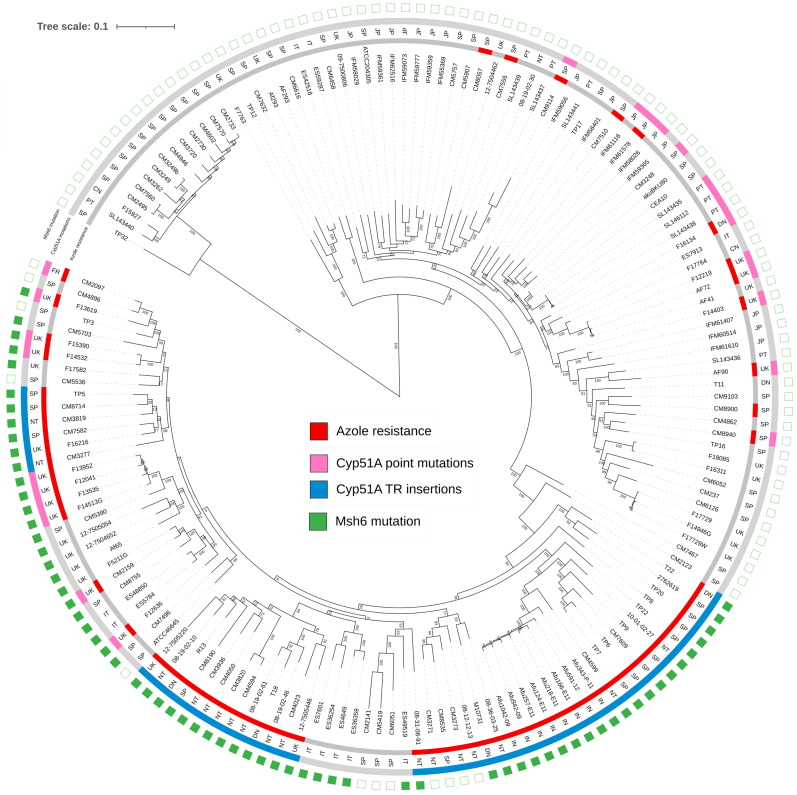
Phylogenetic tree derived from whole genome sequencing of 163 *A. fumigatus* strains. In blue, strains with TR_34_/L98H mutations in Cyp51A; in green, Msh6 mutation (G240A).

We previously performed a whole genome sequencing analysis using a collection of 163 *A. fumigatus* genomes, from which a phylogenetic tree was built [8]. In that analysis, the strains were grouped in four different clusters in which azole-resistant strains were located in clusters I and II (Figure 2).

Azole-resistant strains with point mutations in *cyp*51A were distributed among clusters I and II, while TR resistance mechanisms were only located in three subclusters within cluster II that only contained TR strains. In the present study, the MMR genes *msh*6, *msh*2, *pms*1, and *mlh*1 were analyzed in the *A. fumigatus* genome collection. The mutations detected in these genes are shown in Table 1, together with the percentage of strains harboring them. The analysis showed that genes *msh*2, *pms*1, and *mlh*1 have low genetic variability and do not seem to correlate with azole drug resistance. Several point mutations were found in all genes. However, most of them were cluster-related and more likely representative of the clade.

Remarkably, a mutation in *msh*6 leading to the substitution of glycine (G) for alanine (A) in position 240 (G240A) of the Msh6 protein was found in 42.8% of the isolates and only present in strains from cluster II. Moreover, almost all the strains harboring the TR_34_/L98H azole resistance mechanism also had the G240A Msh6 mutation (Figure 2).

### 3.2. Construction and Phenotypic Characterization of A. fumigatus Δmsh6 Strains

As such a high proportion of the G240A–Msh6 mutation was detected (42%), mainly in azole-resistant strains, we decided to investigate the potential contribution of *msh*6 to the development of drug resistance in *A. fumigatus*. To this end, we deleted the entire *msh*6 gene in the *aku*B^KU80^ strain. To eliminate the possibility of secondary mutations occurring during the construction of the knockout strains, we selected two independent biological replicate transformants to pursue all our phenotypic analyses (strains T3 and T6). To confirm the *msh*6 gene deletion, the region was amplified and sequenced, confirming that the *ptrA* gene had replaced the *msh*6 gene correctly. Both ∆*msh*6 mutants were morphologically indistinguishable from the parental strain regarding macroscopic and microscopic morphology as well as colony radial growth), demonstrating that *msh*6 is not required for normal growth.

### 3.3. Deletion of msh6 Does Not Influence A. fumigatus Azole Susceptibility

E-tests were performed using ITC, VCZ, and PSZ in both the ∆*msh*6 and the *aku*B^KU80^ strains (Figure S1). Microdilution susceptibility assays were also performed with ITC, VCZ, PSZ, and isavuconazole (IVZ). There was no difference in the MIC profile of the ∆*msh*6 strains when compared with the parental strain (Table 2), showing that the deletion of *msh*6, in itself, has no direct impact on azole susceptibility.

### 3.4. Deletion of msh6 Does Not Influence A. fumigatus Virulence

To determine whether there were any differences in virulence between the WT strain and Δ*msh*6 mutants, the *Galleria mellonella* alternative model of infection was used.

**Figure 3:**
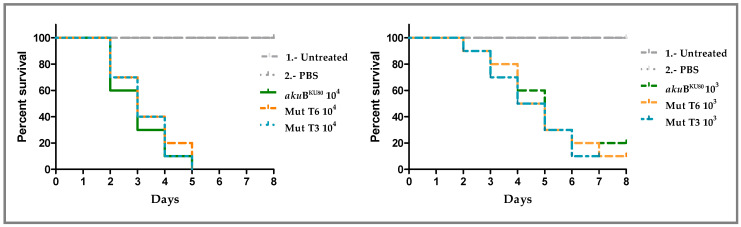
Survival curves of *G. mellonella* larvae inoculated with *aku*B^KU80^ and the ∆*msh*6 (T3 and T6) *A. fumigatus* strains. Using 10^4^ (**Left Panel**) and 10^3^ (**Right Panel**) conidia/larvae inocula. Twenty larvae per isolate and group were used. Control groups include untreated larvae and larvae inoculated only with PBS. The experiment was repeated independently three times.

Using two different infection inocula, we observed that the mortality caused by both Δmsh6 mutants (T3 and T6) was the same as the mortality caused by the parental strain *aku*B^KU80^ (Figure 3). The inoculum of 10^4^ conidia/larvae caused 100% mortality by day 5 post-infection for all strains (Figure 3 Left panel), and the inoculum of 10^3^ conidia/larvae caused 80–90% mortality by day 8 post-infection for all strains (Figure 3 Right panel). The *A. fumigatus* Δ*msh*6 mutants had similar virulence compared to their *akuB*^KU80^ parental strain with both inocula.

### 3.5. Deletion of msh6 Does Not Influence A. fumigatus Growth in Different Stress Conditions

Strains were subjected to growth at a range of different temperatures (37 °C, 45 °C, 50 °C and 60 °C), showing no differences between the parental and the ∆*msh*6 strains. Strains were also subjected to oxidative stress with menadione at 0.04 mM and 0.05 mM, as well as cell wall stress with Congo Red at 10–25–50 µg/mL and Calcofluor white at 10–100 µg/mL. No differences were observed in any of the conditions tested.

### 3.6. Effects of Δmsh6 Deletion on A. fumigatus Antifungal Resistance Development

#### 3.6.1. Development of Resistance to Azole Drugs

A total of 109 conidia of the parental strain (*aku*B^KU80^) and the ∆*msh*6 strains (T3 and T6) were plated on high concentrations of three azole drugs: VCZ, PSZ, and PRZ. Derived mutant colonies were only found on MM + 2 mg/L PSZ. There were no significant differences in the number of mutants obtained from each ∆*msh*6 independent mutant (T3: 4.8 +/− 2.08; T6: 2.66 +/− 0.58) and the parental strain (3.75 +/− 4.5) in three independent experiments (*p* = 0.8, one way ANOVA con Dunnett’s multiple comparisons). No mutants grew on any plate containing VCZ or PRZ.

#### 3.6.2. Development of Resistance to Non-Azole Fungicides

Conidia of the parental strain (*aku*B^KU80^) and both ∆*msh*6 strains (T3 and T6) were plated on high concentrations of three non-azole fungicides commonly used in crop protection; they all belong to different antifungal families and target different genes.

**Figure 4:**
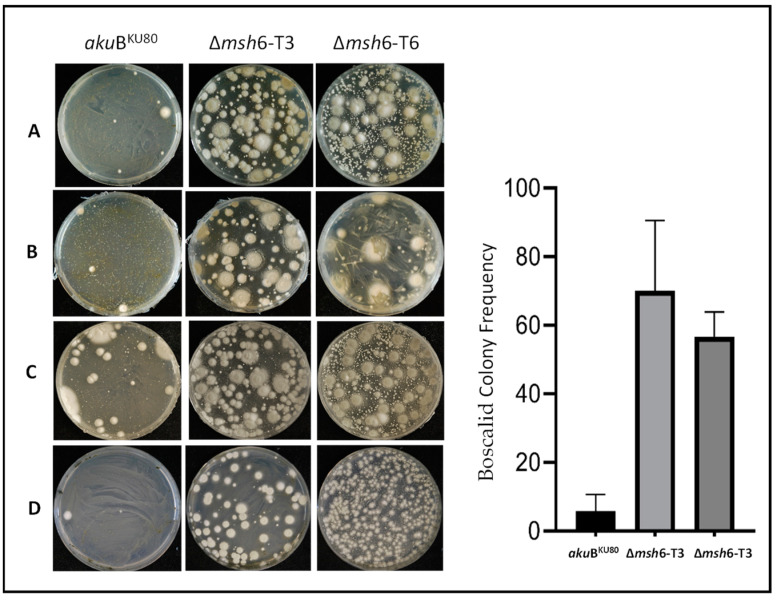
(**Left panel**) *aku*B^KU80^ and ∆*msh*6 mutant strains (1  ×  10^9^ conidia) after 32 mg/L BCL exposure in MM incubated for 7 days at 37 °C. Four independent experiments (**A**–**D**). (**Right panel**) CFUs grown in boscalid-supplemented media. *p* < 0.001 (one-way ANOVA with Dunnett’s multiple comparisons).

BCL was selected as the representative drug for SDHIs, BNY for MBCs, and AZB for QoIs. Deletion of *msh*6 in *A. fumigatus* led to the growth of significantly more resistant colonies after selection on BCL (WT: 5.8 +/− 4.85; T3: 70.1 +/− 20.48; T6: 56.6 +/− 7.23) and BNY (WT: 5.3 +/− 2.31; T3: 12 +/− 5.65; T6: 25 +/− 5.29). Antifungal selection revealed that the Δ*msh*6 strains generated 10- and 2-fold more BCL- and BNY-resistant mutants, respectively, when compared with the parental strain (Figure 4). No resistant mutants were obtained on AZB.

### 3.7. Genetic Analysis of Drug Target Genes

Analysis of the *cyp*51A, *ben*A, and *shd*B polymorphisms and amino acid substitutions found in our chosen set of 123 *A. fumigatus* mutant strains are shown in Table 3. All PSZ mutants were subjected to PCR amplification and sequencing of *cyp*51A. Cyp51A analysis revealed the substitution of glycine (G) in position 54 for tryptophan (W) in 55 strains and arginine (R) in 1 strain. Both mutations have been previously associated with high resistance to long-tailed azole drugs, such as PSZ [45,46].

Over 99% of boscalid selected colonies displayed a high level of resistance to BCL (MICs > 32 μg/mL), and 100% of them (37/37) had a mutation in the target gene (Table 3). PCR amplification and sequencing of these 37 BCL-resistant isolates revealed an amino acid substitution in SdhB of histidine (H) in position 270 for leucine (L) in 12 strains, tyrosine (Y) in 17 strains, and arginine (R) in 8 strains, which seems to be associated with resistance to BCL.

All benomyl-selected colonies displayed a high level of resistance to BNY (MICs > 32 μg/mL). PCR amplification and sequencing of the *ben*A gene were performed for all 30 *A. fumigatus* BNY-resistant strains, showing substitutions of a glutamic acid (E) for six different amino acids, including alanine (A) in one strain, lysine (K) harbored by eight strains, aspartic acid (D) in six strains, valine (V) in one strain or glycine (G) harbored by two strains. Lastly, the amino acid substitution of a phenylalanine (F) for a serine (S) in position 200 was present in 12 isolates.

## 4. Discussion

The worldwide emergence of *A. fumigatus* azole resistance is an important concern in the management of pathologies caused by this important fungal pathogen [3,9,47]. In the agricultural setting, *A. fumigatus*, together with other fungal plant pathogens, are exposed to the selective pressure of DMI drugs and to non-azole fungicides such as QoIs, SDHIs, or MBCs, used to protect crops. In this setting, multiresistant fungal strains are presumably being selected due to mutations on their respective targets, *cyp*51A, *cyt*B, sdhB, and *ben*A genes [8,14,15,16,17]. Based on a previous *A. fumigatus* WGS analysis [8], we found that all genomes harboring the TR_34_/L98H Cyp51A alleles were grouped into three sub-clusters that included resistance to non-azole fungicides. We hypothesized that genome instability could be a potential cause for the development of azole resistance in *A. fumigatus*, related to the phenomenon of multi-drug resistance, as has been suggested before [19,20]. One of the pathways that recognize and repair mistakes and instabilities during DNA replication is the MMR system [26,48], a well-conserved pathway among the phylum *Ascomycota* [29]. It has been reported that deficiencies in MMR genes alter the proper function of this pathway, leading to errors in the reparation of mutations during cell replication [21]. In this study, we investigated four *A. fumigatus* MMR genes, *msh*2, *msh*6, *pms*1, and *mlh*1, to evaluate their possible implication in genetic instability and, thus, their potential contribution to the development of antifungal resistance. Among the MMR genes selected, *msh*6 stands out as the gene that harbors the most prevalent mutation, G240A, present in 42.8% of the strains analyzed. We observed *msh*6 variants in clinical and environmental *A. fumigatus* isolates investigated, which contrasts with the 18.2% *msh*2 variants found in *A. fumigatus* clinical isolates reported previously [20]. Interestingly, we observed that all *msh*6 variants grouped together within Cluster II and, moreover, most of these strains also harbored a TR_34_/L98H azole resistance mechanism and several mutations leading to non-azole fungicide resistance [8]. Based on this, we speculated that mutations in *msh*6 could contribute, to some extent, to the development of antifungal drugs’ multiresistance.

To evaluate the contribution of Msh6 with mutation development we deleted the *msh*6 gene in an *A. fumigatus* azole susceptible wild-type strain. Two ∆*msh*6 independent transformants, T3 and T6, were phenotypically characterized and compared to their parental strain (*aku*B^KU80^). The phenotypic characterization performed did not disclose any difference in growth under various cell stresses, antifungal azole susceptibility, or virulence in the alternative *Galleria mellonella* animal model of infection. These results contrast with those previously obtained for *A. fumigatus msh*2 null mutants, which showed a reduction in virulence compared to the same parental strain [20]. However, our results are in consonance with studies in *Candida glabrata* and *Cryptococcus neoformans*, which demonstrated levels of colonization and infection of *msh*2 null mutants similar to those of the corresponding wild-type strains [21,49,50].

The deletion of *msh*6 does not seem to cause a decrease in fitness. On the other hand, the abundance of strains found with mutations in *msh*6 among clinical strains suggests that these changes could provide some benefits. Therefore, the potentially increased adaptative capacity to different situations, such as antifungal exposure or host immune system attack, could provide a benefit in certain conditions [28,51,52,53,54]. Because all *msh*6 mutated strains were located in a specific cluster, we decided to investigate if an alteration of the MMR pathway functions could derive in a mutator phenotype with an increased mutation frequency in comparison with their parental strain [30].

Since the development of antifungal resistance involves a major problem in the management and treatment of fungal infections, the implication of hypermutator strains has been studied in different human pathogens, as well as the induction of antifungal resistance in in vitro conditions [20,55]. To evaluate the implications of the *msh*6 deletion in antifungal resistance development, we grew Δ*msh*6 isolates and their parental strain (*aku*B^KU80^) under the pressure of azole and non-azole fungicides. Azole drug selection was derived in mutant colonies that grew only on agar plates supplemented with PSZ and not with VCZ or PCZ. However, the number of mutated isolates originating from *msh*6 null mutants did not differ significantly from those of the wild-type strain. Previous studies in other fungal pathogens showed mixed results regarding the implication of MMR genes in the development of azole resistance. In *Candida albicans*, the lack of MSH2 protein has been associated with an increment in the frequency of fluconazole-resistant colony isolation [23]. However, the role of mutations in MSH2 in *C. glabrata* remains unclear, considering that several studies did not find an association between *C. glabrata* isolates harboring MSH2 mutations and the acquisition of azole or echinocandin resistance [50,56]. Nevertheless, other studies have reported the possible promotion of antifungal resistance due to the presence of mutations in MSH2 in *C. glabrata.* This could be possible if the MMR performed a limited and specific role in the acquisition of resistance to antifungals, as is the case of *C. glabrata* [49]. Concerning the genus *Cryptococcus*, a study on MMR in *C. neoformans* demonstrated that the deficiency of MSH2, MLH1, and PMS1 triggered a faster development of resistance to antifungals, as fluconazole-resistant colonies were isolated. However, the mechanisms of genetic stability in relation to azole resistance development have only started to be studied in *A. fumigatus*. To date, in the MMR pathway only Msh2 has been investigated, suggesting that the genetic instability caused by Δ*msh*A mutations can confer an adaptive advantage by increasing posaconazole resistance and virulence acquisition [20]. These same authors, exploring additional DDR mechanisms, have demonstrated the effect of deleting two kinases (*atm*A and *atr*A), conferring an advantage towards the intensity of voriconazole resistance acquisition [19]. Regardless of their individual implications in *A. fumigatus*, it still remains unknown if DNA damage repair and azole resistance development exist in a dynamic and interconnected network [57].

In our study, the analysis of mutation frequencies of *A. fumigatus ∆msh6* strains showed that the loss of *msh*6 results in an increase in mutation numbers in genes that confer resistance to the non-azole fungicides BCL and BNY. The Δ*msh*6 mutants produced 10 times more BCL-resistant isolates and two times more BNY-resistant isolates than the parental strain. Apart from the increased mutated frequencies obtained under this non-azole fungicide, a greater variety of amino acid substitutions were selected. Under BNY pressure, the most common β-tubulin *A. fumigatus* mutations, E198A/Q/and F200Y, were identified in the selected mutants, as well as other mutations such as E198K/D/V/G and F200S that have never been reported in *A. fumigatus* environmental isolates [8,11,12]. Similarly, under BCL pressure, the common H270Y/R *sdh*B field mutations were identified in the laboratory mutants, together with H270S that have never been reported in *A. fumigatus* environmental isolates [8,11]. This finding is in consonance with previous studies that report that laboratory mutagenesis generates a wide variety of mutations, of which only a subset will be reported in the field [58].

However, we did not find mutants resistant to azole drugs other than posaconazole, which makes us wonder whether this finding could be related to the conditions of the mutagenesis assay (very different from what happens in nature) or to additional mechanisms necessary to develop other mutations related to the azole drugs resistance. In addition, the multiple mutations in promoter and coding sequences of *cyp*51A responsible for multi-azole resistance (TR_34_/L98H or TR_46_/Y121F/T289A) likely occur in natural environments in a two or three-step sequential process, as it is known that single mutations (TR_34_ or L98H separately and TR_46_ or Y121F separately) slightly increase the MIC but do not confer complete resistance [44,59]. Therefore, it would not be possible to isolate these mutants in one step with the high level of drug used.

## 5. Conclusions

The results obtained in this study support the hypothesis that the acquisition of antifungal resistance in *A. fumigatus* could at least partially be driven by the genetic instability caused by alterations in the MMR. This hypothesis is also supported by similar findings reported in other fungal species, including *A. fumigatus* [20,21,23,49]. Hence, all evidence suggests that an *A. fumigatus* hypermutator phenotype could provide an advantage in drug-exposed environments without being detrimental to its fungal fitness or virulence.

## Figures and Tables

**Table 1:** Mutations detected in the WGS analysis of *msh*6, *msh*2, *pms*1, and *mlh*1 and the percentage of strains harboring them. Mutation G240A, in bold, was the most prevalent.

Gene (Gene Code)	Mutations	% of Strains
*msh*6 (Afu4g08300)	A55V	0.62
	V118A	0.62
	D121E	0.62
	G178A	1.86
	I183R	10.56
	**G240A**	**42.86**
	N289S	2.48
*msh*2 (Afu3g09850)	A45T	3.73
	P329T	3.73
	E467D	0.62
	E812G	1.24
	A889E	0.62
*pms1* (Afu2g13410)	G286C	0.62
	P401A, V438A, K464R, Q611E, E87K, E760K	4.35
	E444G	2.48
	S758Y	1.24
	D1013Y	0.62
*mlh1* (Afu5g11700)	K310R	4.35
	S368N	4.35
	I510T	1.86
	A641S	4.35

**Table 2:** MIC ranges (EUCAST) to azole drugs for *aku*B^KU80^ and ∆*msh*6 *A. fumigatus* strains.

Strains	MIC Ranges (mg/L)			
	ITC	VCZ	PSZ	IVZ
*aku*B^KU80^	1–1	0.5–0.5	0.125–0.25	1–1
∆*msh*6 T3	0.5–1	0.5–1	0.125–0.25	0.5–1
∆*msh*6 T6	0.5–1	0.5–0.5	0.25–0.25	1–1

**Table 3:** Analysis of the *cyp*51A, *ben*A, and *shd*B polymorphisms and amino acid substitutions found in our set of 123 *A. fumigatus msh*6 mutant strains. Only polymorphisms involving non-synonymous mutations are shown. In bold, amino acid substitutions that have been previously found in resistant strains.

Gene	Nucleotide Position (cDNA)	Codon	Amino Acid Change	*aku*B^KU80^	∆*msh*6-T3	∆*msh*6-T6
***cyp*51A**	g160t	Ggg/Tgg	**G54W**	16	31	80
	g160a	Ggg/Agg	**G54R**	0	1	0
***ben*A**	a593c	gAg/gCg	**E198A**	0	0	1
	g592c	Gag/Cag	**E198Q**	0	0	0
	g592a	Gag/Aag	E198K	0	8	0
	g594c	gag/gaC	E198D	0	3	3
	a593c	gAg/gTg	E198V	0	1	0
	a593g	gAg/gGg	E198G	2	0	0
	t599a	tTc/tCc	F200S	0	10	2
	t599a	tTc/tAc	**F200Y**	0	0	0
***sdh*B**	a809t	cAc/cTc	H270L	0	9	3
	c808t	Cac/Tac	**H270Y**	3	7	7
	a809g	cAc/cGc	**H270R**	0	8	0

## Data Availability

Data are contained within the article and Supplementary Materials.

## Supplementary Materials

The following supporting information can be downloaded at: https://www.mdpi.com/article/10.3390/jof10030210/s1. Figure S1. Azole susceptibility tests (E-tests) were performed on the *aku*B^KU80^ strain and the ∆*msh*6 *A. fumigatus* strains T3 and T6. Table S1. Origin, year of isolation, and geographical origin of the strains used in the NGS analysis. Mutations of the genes described in this work; Table S2. Primers for the construction of the Δ*msh*6 deletion cassette; Table S3. Primers used for sequencing of the Δ*msh*6 deletion cassette.
