# Supplementary material for: Importance of the Aspergillus fumigatus Mismatch Repair Protein Msh6 in Antifungal Resistance Development
Source: J Fungi (Basel). 2024 Mar 12;10(3):210. doi: 10.3390/jof10030210 (PMC10971332; doi:10.3390/jof10030210)

Table S1.- Origin, year of isolation and geographical origin of the strains used in the NGS analysis. Mutations of the genes described in this work.

STRAIN	Cyp51A	CytB	B-TUB	SDH2	Msh6	Country	Origin	Year	CLUSTER
ES42518	WT		WT	WT	--	IT	VET	2016	
CM6616	WT		WT	WT	--	SP	CL	2012	
CM6458	WT		WT	WT	--	SP	CL	2011	
ES59287	WT		WT	WT	--	IT	VET	2016	
09-7500806	WT		WT	WT	--	UK	CL	--	
ATCC204305/2580	WT		WT	WT	--	Ref	--	--	
IFM58029	WT		WT	WT	--	JP	CL	--	
IFM59361	WT		WT	WT	--	JP	CL	--	
IFM62516	WT		WT	WT	--	JP	CL	--	
IFM55369	WT		**F200Y**	WT	--	JP	CL	--	
IFM59359	WT		WT	WT	--	JP	CL	--	
IFM59777	WT		WT	WT	--	JP	CL	--	
IFM59073	WT		WT	WT	--	JP	CL	--	
CM5757	WT		WT	WT	--	SP	CL	2009	
CM5907	WT		WT	WT	--	SP	CL	2009	
12-7504462	WT		WT	WT	--	UK	CL	--	
CM7555	WT (R)		WT	WT	--	SP	CL	2014	
SL143439	WT		WT	WT	--	PG	--	--	
08-19-02-30	WT		WT	WT	--	NT	ENV	--	
CM9114	G54R		WT	WT	--	SP	CL	2017	
SL143437	WT		WT	WT	--	PG	CL	--	
SL143441	WT		WT	WT	--	PG	--	--	
IFM59056	WT		WT	WT	--	JP	CL	--	
IFM58401	WT		WT	WT	--	JP	CL	--	
TP17	WT		WT	WT	--	SP	CL	2008	
CM7510	WT (R)		WT	WT	--	SP	CL	2014	
CBS CA10	WT		WT	WT	--	Ref	CL	--	
akuB^{KU80}	WT		WT	WT	--	Ref	CL	--	
SL143435	N248K		WT	WT	--	PG	--	--	
SL143438	N248K		WT	WT	--	PG	--	--	
SL146112	N248K		WT	WT	--	PG	--	--	
IFM61118	N248K		WT	WT	--	JP	CL	--	
IFM61578	P216L		WT	WT	--	JP	CL	--	
IFM58026	N248K		WT	WT	--	JP	CL	--	
CM3248	N248K		WT	WT	--	SP	CL	2005	
IFM59365	WT		WT	WT	--	JP	CL	--	
F16134	M220K		WT	WT	--	DN	CL	--	
ES7913			WT	WT	--	IT	VET	2017	
F17764	WT		WT	WT	--	CN	CL	--	
F12219	G54R		WT	WT	--	UK	CL	--	
AF41	WT		WT	WT	--	UK	CL	--	
AF72	G54E		WT	WT	--	UK	CL	2002	
F14403	G54R		WT	WT	--	UK	CL	--	
IFM60514	WT		WT	WT	--	JP	CL	--	
IFM61407	WT		WT	WT	--	JP	CL	--	
IFM61610	WT		WT	WT	--	JP	CL	--	
AF90	M220V		WT	WT	--	UK	CL	1997	
SL143436	WT		WT	WT	--	PG	--	--	
T11	WT		WT	WT	--	DN	ENV	2009	
CM8900	WT (R)		WT	WT	--	SP	CL	2017	
CM9103	WT		WT	WT	--	SP	CL	2017	
CM4862	WT		WT	WT	--	SP	CL	2007	
TP16	WT		WT	WT	--	SP	ENV	2013	
CM8940	G54A		WT	WT	--	SP	CL	2017	
F16311	WT		WT	WT	--	UK	CL	--	
F18085	WT		WT	WT	--	UK	CL	--	
CM237	WT		WT	WT	--	UK	CL	--	
CM6126	WT		WT	WT	--	SP	CL	2010	

CM6052	WT		WT	WT	**G240A**	SP	CL	2010	
F17729	WT		WT	WT	--	UK	CL	--	
F17729W	WT		WT	WT	--	UK	CL	--	
F14946G	WT		WT	WT	--	UK	CL	--	
CM7467	WT		WT	WT	--	SP	CL	2014	
CM2123	WT		WT	WT	--	SP	CL	2002	
T22	TR34/L98H		**F200Y**	R51G	**G240A**	DM	ENV	2009	
TP20	TR34/L98H		**F200Y**	WT	**G240A**	SP	CL	2010	
2762619	TR34/L98H		**F200Y**	WT	**G240A**	SP	CL	2010	
TP8	TR34/L98H		**F200Y**	**H270R**	**G240A**	SP	CL	2013	
TP22/90812	TR46/F121Y	**G143A**	**F200Y**	**H270Y**	**G240A**	SP	CL	--	
10-01-02-27	TR34/L98H	**G143A**	**F200Y**	**H270Y**	**G240A**	NT	CL	--	
TP9	TR34/L98H		**F200Y**	WT	**G240A**	SP	CL	2013	
CM7609	TR34/L98H	**F129L**	**F200Y**	WT	**G240A**	SP	CL	2014	
TP7	TR34/L98H		**F200Y**	**H270R**	**G240A**	SP	CL	2013	II
TP6	TR34/L98H		**F200Y**	**H270R**	**G240A**	SP	CL	2012	
CM4599	TR53	**G143A**	**F200Y**	WT	**G240A**	NT	CL	2006	
Afu343-P-11	TR34/L98H		**F200Y**	WT	**G240A**	IN	CL	--	
Afu591-12	TR34/L98H	**G143A**	**F200Y**	WT	**G240A**	IN	CL	--	
Afu218-E11	TR34/L98H	**G143A**	**F200Y**	WT	**G240A**	IN	ENV	--	
Afu166-E11	TR34/L98H	**G143A**	**F200Y**	WT	**G240A**	IN	ENV	--	
Afu124-E11	TR34/L98H	**G143A**	**F200Y**	WT	**G240A**	IN	ENV	--	
Afu257-E11	TR34/L98H	**G143A**	**F200Y**	WT	**G240A**	IN	ENV	--	
Afu1042-09	TR34/L98H	**G143A**	**F200Y**	WT	**G240A**	IN	CL	--	
Afu942-09	TR34/L98H	**G143A**	**F200Y**	WT	**G240A**	IN	CL	--	
08-36-03-25	TR34/L98H + *		**F200Y**	WT	**G240A**	NT	CL	--	
CM8022*	TR34/L98H + *		**E198Q**	**T298A**	**G240A**	SP	CL	2016	
M10731*	TR34/L98H + *		**E198Q**	WT	**G240A**	DN	CL	--	
08-12-12-13	TR34/L98H + *		**F200Y**	WT	**--**	NT	CL	--	
CM3271*	TR34/L98H + *		**F200Y**	WT	--	NT	CL	2004	
CM3273*	TR34/L98H + *		**E198Q**	WT	--	NT	CL	2004	
CM8535	TR34/L98H	**G143A**	**F200Y**	WT	--	SP	CL	2016	
08-31-08-91	TR34/L98H		**F200Y**	WT	**G240A**	NT	CL	--	
ES49519	WT		WT	WT	**G240A**	IT	VET	2016	II
CM6051	WT		WT	WT	--	SP	CL	2010	
CM2141	WT		WT	WT	**G240A**	SP	CL	2002	
CM5419	WT		WT	WT	--	SP	CL	2008	
ES7601	WT		WT	WT	**G240A**	IT	VET	2017	
ES36254	WT		WT	WT	**G240A**	IT	VET	2017	
ES36359	WT		WT	WT	**G240A**	IT	VET	2017	
ES4649	WT		WT	WT	**G240A**	IT	VET	2017	
CM4023	TR34/L98H		WT	WT	**G240A**	NT	CL	2006	
12-7505446	TR34/L98H		WT	WT	**G240A**	UK	CL	--	
08-19-02-46	TR34/L98H		WT	WT	**G240A**	NT	ENV	--	
08-19-02-61	TR34/L98H		WT	WT	**G240A**	NT	ENV	--	
T18	TR34/L98H		WT	WT	**G240A**	DN	ENV	2009	
CM3820	TR34/L98H		WT	WT	**G240A**	NT	CL	2005	
CM4594	TR34/L98H		WT	WT	**G240A**	NT	CL	2004	
CM3936	TR34/L98H		WT	WT	**G240A**	NT	CL	2005	II
CM4050	TR34/L98H		WT	WT	**G240A**	NT	CL	2006	
CM8190	TR34/L98H		WT	WT	**G240A**	SP	CL	2016	
R13	TR34/L98H		WT	WT	**G240A**	DM	ENV	2009	
12-7505220	TR34/L98H		WT	WT	**G240A**	UK	CL	--	
08-19-02-10	TR34/L98H		WT	WT	**G240A**	NT	EN	--	
CM7496	WT		WT	WT	**G240A**	SP	CL	2014	
ATCC46645	WT		WT	WT	--	Ref	--	--	
F12636	G54E		WT	WT	**G240A**	UK	CL	--	
ES48850	WT		WT	WT	**G240A**	IT	VET	2017	
ES5784	WT		WT	WT	**G240A**	IT	VET	2017	
CM8755	WT		WT	WT	**G240A**	SP	CL	2016	II
F5211G	WT		WT	WT	**G240A**	UK	CL	--	
CM2159	M220K		WT	WT	**G240A**	UK	CL	--	
Af65	WT		WT	WT	**G240A**	UK	CL	--	

12-7504652	WT		WT	WT	**G240A**	UK	CL	--	II
12-7505054	WT		WT	WT	**G240A**	UK	CL	--	
CM5390	WT		WT	WT	**G240A**	SP	CL	2008	
F14513G	G138C		WT	WT	**G240A**	UK	CL	--	
F13535	G138C		WT	WT	**G240A**	UK	CL	--	
F13952	G138C		WT	WT	**G240A**	UK	CL	--	
F12041	G138C		WT	WT	**G240A**	UK	CL		
CM3277	TR34/L98H		**E198A**	WT	**G240A**	NT	CL	2007	II
CM7582	TR34/L98H		**E198A**	WT	**G240A**	SP	CL	2014	
F16216	TR34/L98H		WT	WT	**G240A**	UK	CL	--	
CM3819	TR34/L98H	**G143A**	**E198A**	WT	**G240A**	NT	CL	2005	
TP5	TR34/L98H		**E198A**	WT	**G240A**	SP	CL	2013	
CM8714	TR34/L98H		**E198A**	WT	**G240A**	SP	CL	2016	
F17582	WT		WT	WT	**G240A**	UK	CL	--	
CM5536	WT		WT	WT	--	SP	CL	2009	
F15390	M220T		WT	WT	**G240A**	UK	CL	--	
F14532	M220T		WT	WT	**G240A**	UK	CL	--	
CM5703	WT		WT	WT	**G240A**	SP	CL	2009	
TP3	WT		WT	WT	--	SP	ENV	2012	
F13619	H147Y, G448S		WT	WT	**G240A**	UK	CL	---	
CM2097	G54R		WT	WT	--	FR	CL	1997	
CM4896	WT		WT	WT	--	SP	CL	2007	
F7763	5SNPs		WT	WT	--	UK	CL	--	III
TP12	5SNPs		WT	WT	--	SP	CL	2001	
CM7632	5SNPs		WT	WT	--	SP	CL	2015	
AF293	5SNPs		WT	WT	--	Ref	CL	--	
TP32	3SNPs		WT	WT	--	SP	CL	2012	IV
F15927	3SNPs		WT	WT	--	CN	CL	--	
SL143440	3SNPs		WT	WT	--	PG	--	--	
CM2495	3SNPs		WT	WT	--	SP	CL	2003	
CM2730	3SNPs		WT	WT	--	SP	CL	2004	
CM2733	3SNPs		WT	WT	--	SP	CL	2004	
CM3249	3SNPs		WT	WT	--	SP	CL	2005	
CM3262	3SNPs		WT	WT	--	SP	CL	2005	
CM3720	3SNPs (R)		WT	WT	--	SP	CL	2006	
CM4602	3SNPs		WT	WT	--	SP	CL	2006	
CM4946	3SNPs		WT	WT	--	SP	CL	2007	
CM7560	3 SNPs		WT	WT	--	SP	CL	2014	
CM7570	3 SNPs		WT	WT	--	SP	CL	2014	

Polymorphisms in Cyp51A: 5SNPS (F46Y/M172V/N248T/D255E/E427K); 3SNPs (F46Y/M172V/D255E). TR34/L98H + * (TR34/L98H/S297T/F495I). Spain, SP; Portugal, PG; Canada, CN; India, IN; United kingdom, UK; Denmark, DN; The Netherlands, NT; France, FR; Japan, JN; Reference, Ref.

Table S2: Primers for the construction of the Δ*msh6* deletion cassette.

Primer name	Sequence (5′-3′)	Amplicon size
p1	AGCTCGACTTCGATACAGAAGCCTCCAATG	1300 pb
p2	GTTACCAATGGGATCCCGTAATCAATTGCCCGTTTAGTGGTGGTATCGGTATGGACACTC	
p3	GAGTGTCCATACCGATACCACCACTAAACGGGCAATTGATTACGGGATCCCATTGTAAC	1900 pb
p5	CATACTCCCACTAGAACCCTTGACGAAGCAATGGCCTCTTGCATCTTTGTTTGTATTATA	
p4	TATAATACAAACAAAGATGCAAGAGGCCATTGCTTCGTCAAGGGTTCTAGTGGGAGTATG	1400pb
p6	CCTTTATCATCGTGCGCCTCCTATTTCTCC	
p7	CCAGTGTGCTACAGCTTACCCAAGTAAGAG	4600 pb
p8	ACGCCCTCTCACTGTCACTATATCTACTGC	

Table S3: Primers used for sequencing of the Δ*msh6* deletion cassette.

Primer name	Sequence (5′-3′)
s1	GCATCCAGTAGACCAGTTTCC
s2	GCGCACAAGCACAGGATCTC
s3	GTGTTCGTTCCCAGTCATCG
s4	CAGAAGGACGAATGCAATATC
s5	CCAGTTGACGACAACACCAGC
s6	CAACCACCTAGATCGACTACG
s7	GTCCATCATAGTTGGTGCGC
s8	GCAACTGCAAGTTCCAAAGC
s9	CAGCGTTCTCATCTTAATGAC
s10	GCTTAGCCTGTCCTTTCTTGG
s11	GGAAGAACGAGAGAAGGCATG
s12	GCTCTTCAATGCTACCGCTG

Supplementary Figure S1

Figure S1. Azole susceptibility tests (E-tests) performed on the *aku*B^{KU80} strain and the Δ*msh6 A. fumigatus* strains T3 (Δ*msh*6) and T6 (Δ*msh*6)

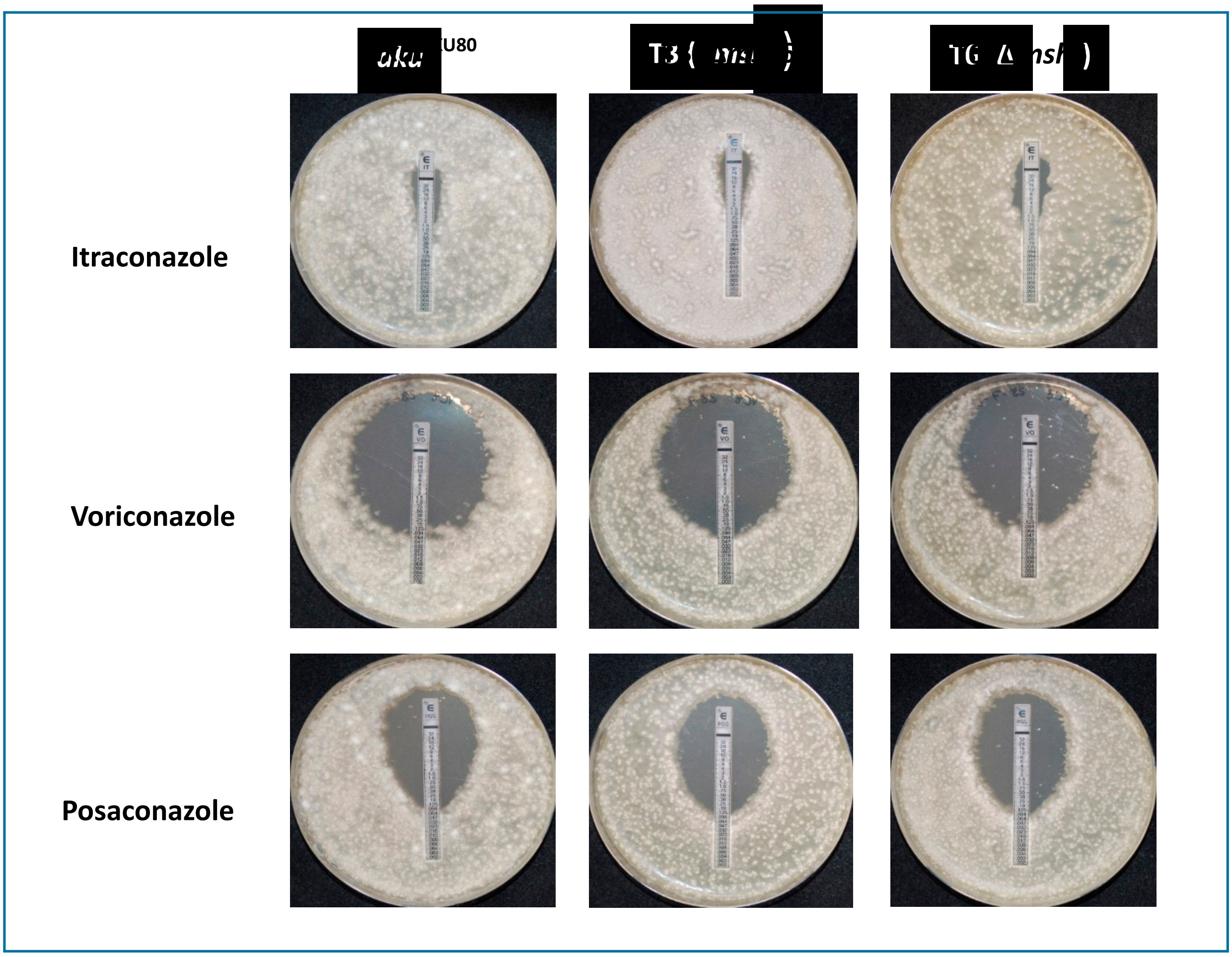

Supplement: Supplementary file 1 [file jof-10-00210-s001.zip › jof-2781556-supplementary.pdf]
